# Synthesis and Structure of a 22 × 12 × 12 Extra-Large Pore Zeolite ITQ-56 Determined by 3D Electron Diffraction

**DOI:** 10.1021/jacs.1c02654

**Published:** 2021-06-02

**Authors:** Elina Kapaca, Jiuxing Jiang, Jung Cho, José L. Jordá, María J. Díaz-Cabañas, Xiaodong Zou, Avelino Corma, Tom Willhammar

**Affiliations:** † Berzelii Centre EXSELENT on Porous Materials, Department of Materials and Environmental Chemistry, Stockholm University, SE-106 91 Stockholm, Sweden; ‡ MOE Key Laboratory of Bioinorganic and Synthetic Chemistry, School of Chemistry, Sun Yat-Sen University, Guangzhou 510275, China; § Instituto de Tecnología Química, Universitat Politècnica de València-Consejo Superior de Investigaciones Científicas, Avenida de los Naranjos s/n, 46022 Valencia, Spain

## Abstract

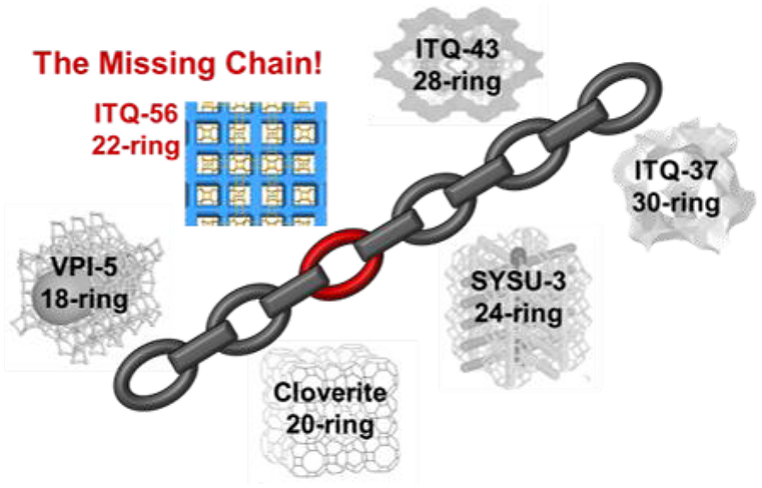

A multidimensional extra-large pore germanosilicate, denoted ITQ-56, has been synthesized by using modified memantine as an organic structure-directing agent. ITQ-56 crystallizes as plate-like nanocrystals. Its structure was determined by 3D electron diffraction/MicroED. The structure of ITQ-56 contains extra-large 22-ring channels intersecting with straight 12-ring channels. ITQ-56 is the first zeolite with 22-ring pores, which is a result of ordered vacancies of double 4-ring (*d*4*r*) units in a fully connected zeolite framework. The framework density is as low as 12.4 T atoms/1000 Å^3^. The discovery of the ITQ-56 structure not only fills the missing member of extra-large pore zeolite with 22-ring channels but also creates a new approach of making extra-large pore zeolites by introducing ordered vacancies in zeolite frameworks.

## Introduction

**Scheme 1:**
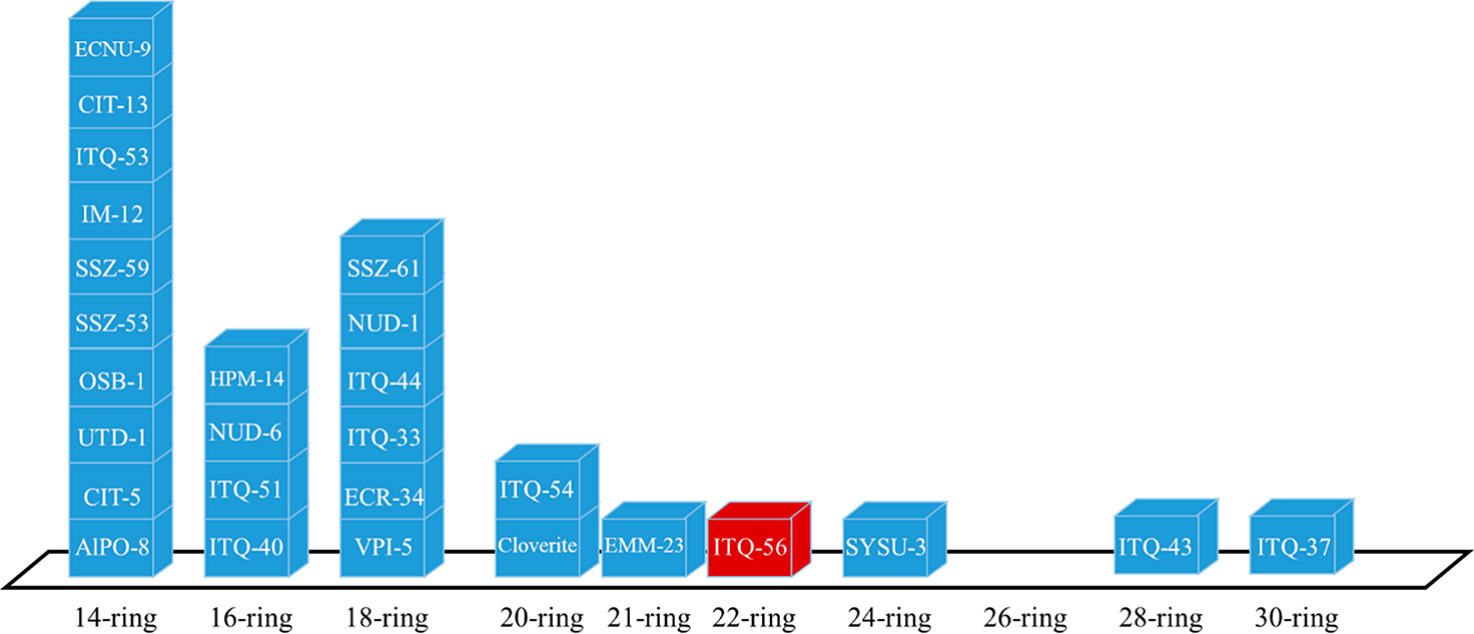
Pore-Size Distribution of Extra-Large Pore Zeolites by Ring Size Opening Discovery of ITQ-56 fills the gap of 22-ring zeolite.

Zeolites are crystalline microporous materials that consist of corner-sharing tetrahedral (TO_4_) units (T = Si, Al, Ge, Ga, P, Be, Ga, B, etc.) and well-defined pore structures on the molecular scale. Zeolites are classified by the number of T atoms delimiting the pore openings as small (8-ring), medium (10-ring), large (12-ring), and extra-large (>12-ring) pore zeolites. They are used in a wide range of applications in catalysis, gas separation, ion exchange, and so on. Great efforts have been made to target synthesis processes that can lead to zeolites with extra-large pores, specific composition,^1^ and functional oriented cavities.^2^ Large efforts have been made to synthesize and characterize extra-large pore zeolites, as summarized in several review articles.^3,4^ One of the strategies was to combine the use of germanium and large/rigid organic structure-directing agents (OSDAs).^5^ To date, this has resulted in more than 10 new zeolite structures. The introduction of germanium prompted the formation of double 4-rings (*d*4*r*s)^6^ promoting the formation of extra-large pores in the structures. Interestingly, the presence of *d*4*rs* in germanosilicate zeolites has created another approach of making new zeolites, that is, via the assembly–disassembly–organization–reassembly (ADOR) approach.^7^ This process is possible due to O–Ge–O bonds that are hydrothermally unstable. With the addition of water or acid, these bonds break to form silica-rich layers, which by organization and reassembly create a new 3D zeolite framework. Zeolite UTL framework topology has been very feasible for this approach, producing a new family of zeolites: IPC-2, IPC-4, IPC-6, IPC-7, IPC-9, and IPC-10.^8^ Wu et al.^9^ summarized the recent progress in the structure stabilization and structure modification of germanosilicates.^10^ In addition to that, they also successfully prepared a 14 × 10-ring extra-large pore zeolite ECNU-9 by interlayer expansion. The synthesis of extra-large pore zeolites has also been realized via the supramolecular assembly templating (SAT) approach.^11^ In this case, organic molecules have played an important role in the formation of supramolecular assemblies that act as OSDAs during zeolite synthesis. Using this approach several new extra-large pore germanosilicates have been synthesized, for example, NUD-1 (18-ring),^12^ NUD-2 (14-ring),^13^ ITQ-37 (30-ring),^14,15^ NUD-5,^16^ and NUD-6.^17^ Recently, Camblor et al. presented an interesting zeolite HPM-14 with an interconnected extra-large and odd small-ring (16 × 9 × 8) channel system.^18^ Despite the great efforts, zeolites with extra-large pores are still rare. The pore openings of all reported extra-large pore zeolites range from 14-ring to 30-ring and are summarized in Scheme 1. It is interesting to note that except for EMM-23, all of the reported extra-large pores zeolites have their largest pores defined by an even number of TO_4_ tetrahedra. Among the six zeolites with pore openings larger than 18-ring, four of them (ITQ-54,^19^ SYSU-3,^20^ ITQ-43,^21,22^ and ITQ-37^14^) contain germanium. Among the extra-large pore zeolites with a ring opening up to 30-ring, most even rings have been occupied, except for 22-ring and 26-ring zeolites, whereas only two zeolites contain odd rings, 15-ring (GeZA)^23^ and 21-ring (EMM-23).^24^

Although germanium is a very good candidate to make extra-large pore zeolites,^25^ many germanium-rich zeolites are synthesized as polycrystalline powders with crystal sizes that are too small (<5 μm) for structure solution by single-crystal X-ray diffraction (XRD).^14^ Powder XRD (PXRD) patterns for zeolite structures with large unit cells contain severe peak overlapping, and hence the structure determination in this case is very challenging.^26^ Electron crystallography, especially the 3D electron diffraction (ED) techniques,^27^ has been shown to be powerful for the structure determination of very complex structures including zeolites.^19,28−30^ Several methods have been developed for 3D ED data collection.^31−33^ A common feature of the 3D ED method is that a crystal in an arbitrary orientation is rotated along the goniometer axis, and patterns are recorded on the crystal at different angles. A more recent approach for the collection of 3D ED data has been developed using continuous crystal rotation, where ED patterns are collected in a movie mode, known as MicroED,^34^ fast ED tomography,^35^ and continuous rotation electron diffraction (cRED).^36^ In such an approach, the total data collection time is only a few minutes or even less. The cRED allows for the collection of almost complete high-quality 3D ED data from very beam-sensitive materials such as germanium-rich zeolites, metal–organic frameworks (MOFs), proteins, and small organic molecules. This provides new opportunities to solve structures that have remained unsolved for decades.^37−39^ The strong interaction between electrons and matter enables the collection of single-crystal data from submicrometer sized crystals. Despite this, the ED data will be influenced by multiple scattering, which will cause high refinement *R*-values for kinematic refinement, and the atomic positions after structure refinement will show high accuracy.^40,41^

In the search for new OSDAs for the synthesis of extra-large pore zeolites, it has been found that the expansion of the size of the OSDAs promotes the formation of zeolites with extra-large pores. For example, just by adding methyl groups to isoindoline-based OSDA, which are used to synthesize 12-ring channel zeolites, and slightly modifying the chemical composition, zeolite ITQ-43 with 28-ring channels can be achieved.^42^ Following the idea of expanding OSDAs, we selected *N*,*N*,*N*-trimethyl-adamantammonium TMA-da^+^, which has been demonstrated to be an effective OSDA for the syntheses of SSZ-13,^43^ SSZ-23,^44^ and ITQ-1,^45^ and we identified memantine (a drug to treat Alzheimer’s disease) with two additional methyl groups compared with adamantamine, as a starting molecule for preparing OSDAs directed to the synthesis of extra-large pore zeolites. Using 3,5,*N*,*N*,*N*-pentamethyl-1-adamantammonium as the OSDA, we synthesized the new germanosilicate zeolite ITQ-56. The structure is featured with *d*4*r* vacancies creating extra-large 22-ring channels that fill the gap of the extra-large pore channel axis.

## Results and Discussion

ITQ-56 was obtained from a hydrothermal synthesis batch Si/Ge ratio of 2:1 using 3,5,*N*,*N*,*N*-pentamethyl-1-adamantammonium as an OSDA (Figure S1). The crystallization was carried out at 200 °C for 1 day under static conditions from a synthesis gel with the composition 0.667SiO_2_/0.333GeO_2_/0.15OSDAOH/0.15NH_4_F/3H_2_O; see the SI for more details. The crystals had a plate-like morphology with a size of ∼2.00 × 0.50 × 0.02 μm (Figure S3). The plate-like crystals were closely packed in large building blocks that were surrounded by an amorphous material.

cRED data were collected from a large number of crystals that showed various qualities depending on the crystals. Six cRED data sets with the best statistics in terms of *R*_int._ were chosen for the structure determination of ITQ-56. The 3D reciprocal lattice and 2D slices from one representative data set are presented in Figure S4. The six cRED data sets were scaled and merged together into one *hkl* list file and used for structure solution and refinement. The merged data set has a completeness of 82% and a resolution up to 1.1 Å and contains 58 235 reflections, among which 2903 are unique (*R*_int._ = 0.233) (Table 1). The structure solution was done by direct methods using SIR2014^46^ software, and refinement was done using SHELXL^47^ software. All T and O atoms were found during the structure solution from individual datasets as well as the merged dataset. 246 parameters were refined using 40 geometric restraints for T–O distances, and the refinement converged with a final *R*1 value of 0.284.

**Table 1:** Continuous Rotation Electron Diffraction (cRED) and Structure Refinement Details of ITQ-56

parameters	
crystal system	orthorhombic
space group	*Immm* (71)
*a* (Å)	13.51
*b* (Å)	26.40
*c* (Å)	55.09
volume (Å^3^)	19648.6
λ (Å)	0.0251
tilt range per frame (deg)	0.23
exposure time per frame (s)	0.5
completeness (%)	82
no. parameters	248
resolution (Å)	1.1
no. restraints	40
*R*_int._	0.233
reflections collected/unique	58235/2903
*R*1	0.286
*wR*_2_	0.560
GoF	2.38

The structure of ITQ-56 has orthorhombic symmetry and is of space group *Immm* (no. 71) with very large unit-cell parameters *a* = 13.5066 Å, *b* = 26.5896 Å, *c* = 55.5013 Å. It has an exceptionally long *c* parameter that is the fourth longest among zeolites published in the Database of Zeolite Structures.^48^ Only zeolites IM-5^49^ (*b* = 57.2368 Å), AlPO-78^50^ (*c* = 60.6099 Å), and SSZ-57^51^ (*c* = 109.7560 Å) contain longer cell dimensions than ITQ-56. The structure has 19 symmetry-independent T atoms, which are all shared by silicon and germanium (the Si/Ge ratio is 1.15 from cRED data), and 49 O atoms per asymmetric unit. The Si/Ge ratio refined from cRED data is lower than that obtained by inductively coupled plasma (ICP), which is due to the presence of Si-rich amorphous material in the sample. Among the 252 zeolite structures in the Database of Zeolite Structures, only 10 have more than 19 T atoms in the asymmetric unit. Interestingly, most of these frameworks are disordered (ITQ-39,^52^ IPC-6,^53^ SSZ-57,^51^ SSZ-61,^54^ SSZ-31 polymorph I,^55^ SSZ-70^56^) or interrupted (ITQ-39,^52^ SSZ-61,^54^ SSZ-74,^57^ SSZ-70^56^).

The Pawley fit of synchrotron PXRD data confirmed the space group *Immm* (no. 71) and unit-cell parameters. The broad peaks and relatively low resolution of the synchrotron PXRD data (intensities start to decay from 2θ of 10°, *d* = 2.3 Å) together with a significant contribution from the amorphous impurity in the sample made the Rietveld refinement of such a complex structure very challenging. The Rietveld refinement was done to locate OSDA molecules and compare the structure to the one achieved with cRED data.

**Figure 1:**
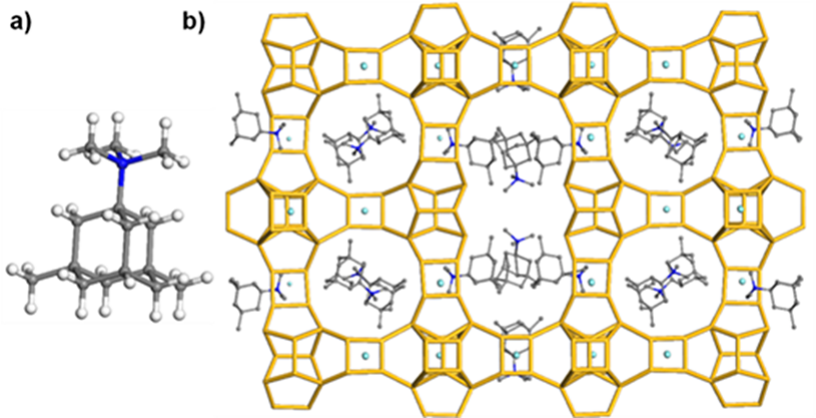
(a) OSDA molecule used for the synthesis of ITQ-56. Carbon atoms are gray, the nitrogen atom is blue, and hydrogen atoms are white. (b) Location of the OSDA molecules in the structure of ITQ-56 viewed along [100] with T atoms in yellow and oxygen atoms omitted for clarity.

The Rietveld refinement (Figure S5, Table 2) converged to *R*_*wp*_ = 0.149 and confirmed the structural model of ITQ-56 obtained using the cRED data. The fluoride ions were located in the middle of *d*4*r*s from the difference Fourier map (Figure S11). The starting positions for the four OSDA molecules (Figure 1a) were obtained from the difference Fourier map obtained from the refinement against cRED data (Figure S5) and later refined against synchrotron PXRD data. During refinement, the position of the OSDA molecules changes slightly because the difference map from cRED data will be altered by multiple scattering and the possible degradation of the OSDA molecule in the electron beam. Their positions were refined using geometric restraints, which were gradually released during the refinement. Four symmetry-independent OSDA molecules are located in the pores of ITQ-56 (Figure 1b). Two of them are located in the 22-ring channel, and two are in the 12-ring channels along [100] and [010], respectively. The chemical analysis of the sample indicates that the OSDA is intact in the pores, with a C/N ratio of 14.9, which is close to the theoretical value of 15. This is further confirmed by the good agreement between the ^13^C liquid NMR of the OSDA and the ^13^C-MAS NMR of ITQ-56 (Figure S7).

**Table 2:** Details of Rietveld Refinement of As-Made ITQ-56

parameters	
crystal system	orthorhombic
space group	*Immm* (71)
*a* (Å)	13.5076
*b* (Å)	26.5882
*c* (Å)	55.4883
λ (Å)	0.39984
temperature (°C)	20
2θ range (deg)	0.75–20.0
resolution (Å)	1.15
no. of restraints	78 for T–O, 120 for O–T–O, 46 T–O–T
no. of reflections	3807
*R*_wp_	0.149
*R*_exp_	0.057
GoF	2.64

The Si/Ge ratio (1.15) after refinement using cRED data is lower than that (1.37) obtained by the Rietveld refinement. The cRED data result is from six individual crystals chosen for the cRED data collection, but the PXRD data show the Si/Ge ratio from the whole crystalline sample and may slightly differ. The ^19^F MAS NMR spectrum (Figure S8) shows a single resonance band at −8.42 ppm, indicating that fluorine anions are trapped in the *d*4*r*s, which is confirmed by Rietveld refinement. The fluoride content from cRED (12.2) compared with that obtained by Rietveld refinement (9.6) also differs. This could be due to the sample inhomogeneity. The structure obtained from cRED data was from six crystals, whereas the Rietveld refinement represents the bulk sample. A comparison between the CHN analysis and the OSDA content obtained from the structure refinement leads to an estimation of 18% amorphous content in the synthesized material. Energy-dispersive X-ray spectroscopy (EDS) shows that the amorphous material is Si-rich. This explains why the Si/Ge ratio in ICP analysis (2.24) is higher than that obtained by the structure refinement of ITQ-56. The thermogravimetric analysis of the as-made ITQ-56 shows a total weight loss of 17.2 wt %, which corresponds to the organic OSDA and fluorine ions occluded within the zeolite (Figure S9).

**Figure 2:**
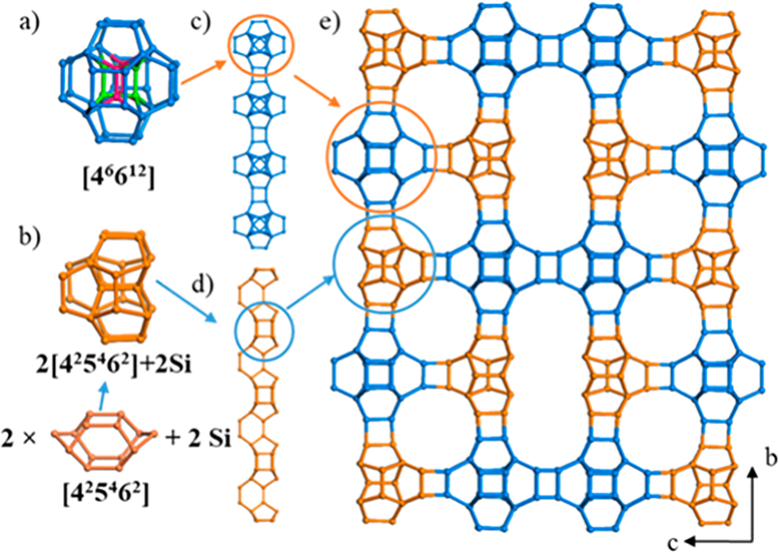
Construction of the ITQ-56 framework. (a) Illustrated [4^6^6^12^] cage with different orientations of 4-rings marked in pink and green. (b) “double” [4^2^5^4^6^2^] cage with one 4-ring in common and two additional TO_4_ dimers at one side. (c) Cage chain of [4^6^6^12^] created by connecting them via *d*4*r*s, in blue. (d) Cage chain of “double” [4^2^5^4^6^2^] connected via two 4-rings, in brown. (e) 3D framework of zeolite ITQ-56 viewed along [100]. Only the connections of T atoms are shown for clarity.

The structure of zeolite ITQ-56 is built from two different cages: a “double” [4^2^5^4^6^2^] cage with one 4-ring in common and two additional dimers at one side and a [4^6^6^12^] cage in which 4-rings at two different orientations with occupancy of 0.5 are located (Figure 2a,b). The same types of the cages are connected to each other via oxygen atoms to form chains along *a* (Figure 2c,d). Two chains containing the same type of cages are paired together along *c*; there is no connection between two “double” [4^2^5^4^6^2^] cages producing terminal T atoms. The paired chains are then shifted by 0.5 along *a*, *b*, and *c* and connected via oxygen atoms to form the 3D structure (Figure 2e).

The structure of ITQ-56 exhibits a 3D channel system with extra-large 22-rings along [100]. There are straight 12-ring channels along [100], ⟨110⟩, and ⟨101⟩. There is also a straight 12-ring channel along [010]. The extra-large 22-ring channel is created due to the nonexistent *d*4*r*s. Furthermore, the channels are also interconnected by channel connections.

**Figure 3:**
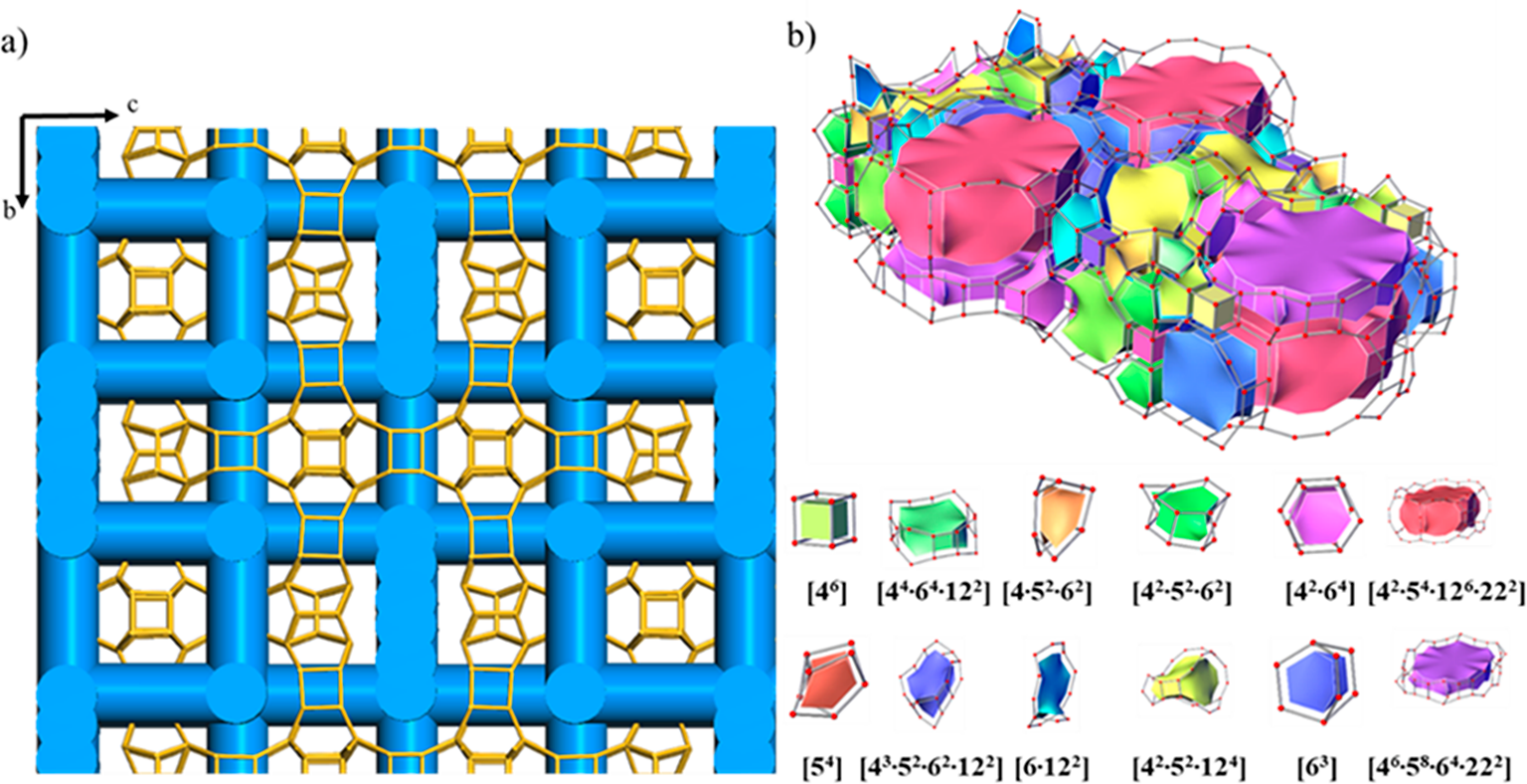
(a) Steric view of the channel configuration, showing the interconnectivity of the 22-ring channels with the 12-ring channels. (b) Illustration of the channel system and cavities in the ITQ-56 by tiling and nets.

The steric view of the channel system is shown in Figure 3a. The pore windows and the sizes of channels are presented in Figure S10. The free diameters of 12-ring pore openings are ∼7 Å, and the extra-large 22-ring has pore sizes of 4.7 Å × 19.9 Å, providing a very large cavity in the structure. The structure is also illustrated by tiling and nets, as shown in Figure 3b. The ITQ-56 exhibits transitivity of (18)(44)(48)(22), in which there are 18 independent vertices (omitting the disorder in the cage of [4^6^6^12^]), 44 independent edges, 48 independent facet classes, and 22 independent tile classes. Among the 22 tile classes, 12 important classes are shown in Figure 3b.

The framework of ITQ-56 is closely related to that of several zeolites that contain 3D 12-ring channels: ITQ-26 (with framework type code IWS,^48^ space group *I*4/*mmm* (no. 139), *a* = 26.7769 Å, and *c* = 13.2505 Å),^58^ ITQ-21 (space group *Fm*3̅*c*, *a* = 27.689 Å),^59^ and PKU-14.^60^ The projection of ITQ-56 along *c* is very similar to that of ITQ-26 along *a* and *b*. The projection of ITQ-56 along *a* and the projection ITQ-26 along *c* vary with a repetition of the two cages: a “double” [4^2^5^4^6^2^] cage with one 4-ring in common and a [4^6^6^12^] cage. For ITQ-26, these cages are alternating, but for ITQ-56, there is a mirror plane perpendicular to the *c*-axis producing two similar cages next to each other. (See Figure S11.) There are also some other similar zeolite structures, for example, ITQ-21 that is built entirely of [4^6^6^12^] cages interconnected via *d*4*r*s. Whereas the structure of ITQ-21 contains a single 4-ring that is disordered, in the structure of PKU-14,^60^ the same 4-ring is missing, and in NUD-3,^61^ the 4-ring appears ordered. The aforementioned zeolites (ITQ-26 and PKU-14) have local disorder in the [4^6^6^12^] cage. In the case of ITQ-26, there are 4-rings with three different orientations inside the cage. The structure of PKU-14 has eight terminal hydroxyl groups, creating a large void that can accommodate a (H_2_O)_2_ dimer. In the case of ITQ-56, the refinement using cRED data shows that there exist two different orientations of the 4-ring with an occupancy of 0.5 each, as shown in Figure 2a.

The solid-state ^29^Si MAS NMR analysis of the as-made sample shows the selective enhancement of the signal at −100.8 ppm with a shoulder at about −93 ppm in the Si cross-polarization (CP) MAS NMR compared with the ^29^Si Bloch decay (BD) MAS NMR spectra (Figure S12). This implies the presence of the Q3 (≡Si–OH) species. Argon adsorption gives a peak centered at 0.71 nm with a shoulder at 0.85 nm that may correspond to the cyclic 12-ring large pore and the elliptical 22-ring extra-large pore, respectively. Nitrogen adsorption measurements gave a BET surface area of 484.2 m^2^/g. The actual BET surface of ITQ-56 is higher owing to the presence of the amorphous solid in the sample and some loss of crystallinity during the calcination process (Figure S13). The crystallinity of ITQ-56 was retained after calcination up to 600 °C under a dry air atmosphere (Figure S14).

**Figure 4:**
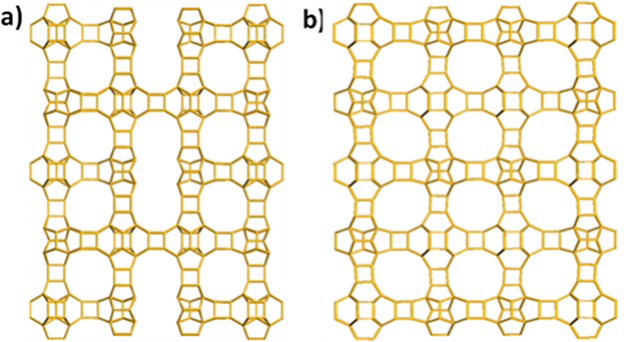
Comparison of (a) the ITQ-56 framework with (b) a hypothetical fully connected zeolite framework deduced from ITQ-56. Only T–T connections are shown for clarity.

The unique extra-large channels with a 22-ring opening in ITQ-56 are formed due to a missing *d*4*r* unit in the center of the 22-ring (Figure 4a). If the missing *d*4*r* unit had been added, then a fully connected geometrically feasible 3D framework with a 3D intersecting 12-ring channel system would have been formed (Figure 4b). The completed version of ITQ-56 shows a similar lattice energy as those of ITQ-7 (ISV), ITQ-21, and ITQ-26 (IWS), indicating that the geometric feasibility of the framework itself is equally good. The large OSDA is plausibly an important factor for the vacant *d*4*r* units and the formation of the extra-large 22-ring channels. In the synthesis of ITQ-21, ITQ-26, and PKU-14 with 3D 12-ring channels, the sizes of the OSDA molecules (*N*(16)-methylsparteinium hydroxide for ITQ-21, 1,3-bis(triethylphosphoniummethyl)-benzene for ITQ-26, and dicyclohexyldimethylammonium hydroxide for PKU-14) are smaller and can be easily packed in the 12-ring channels. Although the computational OSDA docking modeling showed that it is possible to pack the used OSDA molecules in a fully connected version of ITQ-56, the stabilization energy is significantly higher (−11.6 kJ/mol T) compared with that for ITQ-56 with the missing *d*4*r* (−32 kJ/mol T). This shows that OSDA packing is favored in ITQ-56 with missing *d*4*r*.

Following a similar pattern as that for ITQ-56, interrupted frameworks with *d*4*r* vacancies were created based on the related structures ITQ-7 (ISV), ITQ-21, and ITQ-26 (IWS); see Figure S15. The calculated lattice energies of these structures were in the same range as those for ITQ-56. (See Table S1.) This indicates that using carefully chosen synthesis conditions, it may also be possible to introduce *d*4*r* vacancies and subsequently generate extra-large 22-ring channels in these materials.

The discovery of ITQ-56 demonstrates a new approach for making extra-large pore zeolites by introducing ordered vacancies in the zeolite framework. The cRED data show no evidence of disorder, indicating that the *d*4*r* vacancies are highly ordered. On the basis of the cRED data, the vacant *d*4*r* was evident, whereas the refinement based on the synchrotron PXRD data did not show any significant difference in the fit for the structure with or without *d*4*r*. This shows the power of single-crystal 3D ED for the elucidation of complex structures including fine structural details such as *d*4*r* vacancies.

## Conclusions

The first zeolite containing extra-large 22-ring channels has been synthesized using modified memantine as the OSDA. The structure of ITQ-56 is complex and has exceptionally large unit-cell parameters. The framework structure of ITQ-56 was determined by cRED, and the OSDAs were located by Rietveld refinement using synchrotron PXRD data. The framework of ITQ-56 contains a 3D 22 × 12 × 12 channel system and is closely related to several zeolites that contain 3D 12-ring channels. We show that highly ordered extra-large pore zeolites can be synthesized by the systematic removal of *d*4*r* units in zeolite frameworks. This indicates a new approach for making novel extra-large pore zeolite frameworks by introducing OSDAs that facilitate the stabilization of ordered vacancies in zeolite frameworks generated by the selective removal of secondary building units, as *d*4*r* units in the present case. The discovery of ITQ-56 fills the gap of the “missing 22-ring chain” among the extra-large pore zeolites.

## Supporting Information Available

Synthesis of OSDA, zeolite ITQ-56, structure analysis by TEM, Rietveld refinement, chemical analysis, textural properties, and energetic studies (PDF)
