# Supplementary material for: Synthesis and Structure of a 22 × 12 × 12 Extra-Large Pore Zeolite ITQ-56 Determined by 3D Electron Diffraction
Source: J Am Chem Soc. 2021 Jun 2;143(23):8713–9. doi: 10.1021/jacs.1c02654 (PMC8213054; doi:10.1021/jacs.1c02654)
Supplement: Supplementary file 1 — ja1c02654_si_001.pdf [file ja1c02654_si_001.pdf]

SUPPORTING INFORMATION

Synthesis and structure of a 22×12×12 extra-large pore zeolite ITQ-56 determined by 3D electron diffraction

Elina Kapaca[a,#], Jiuxing Jiang[b,#], Jung Cho[a], José L. Jordá[c], María J. Díaz-Cabañas[c], Xiaodong Zou[a], Avelino Corma[c,*], Tom Willhammar[a,*]

[a] Berzelii Centre EXSELENT on Porous Materials, Department of Materials and Environmental Chemistry, Stockholm University, SE-106 91 Stockholm, Sweden

[b] MOE Key Laboratory of Bioinorganic and Synthetic Chemistry, School of Chemistry, Sun Yat-Sen University, Guangzhou 510275, China

[c] Instituto de Tecnología Química, Universitat Politècnica de València-Consejo Superior de Investigaciones Científicas, Avenida de los Naranjos s/n, 46022 Valencia, Spain

*acorma@itq.upv.es, tom.willhammar@mmk.su.se

Synthesis of OSDA 3
Synthesis of ITQ-56 zeolite 3
Structural analysis by transmission electron microscope (TEM) and continuous rotation electron diffraction (cRED). 3
Rietveld refinement....4
ICP analysis..4
CHN analysis..4
Thermogravimetric analysis 4
Temperature depended in situ PXRD 4
Scanning electron microscopy (SEM)..5
NMR studies 5
Textural properties...5
Energetic studies..5
Lattice energy 5
Stabilization energy 6
Figure S1: *Structure of OSDA used for synthesizing the ITQ-56 zeolite.*.................................6
Figure S2: *Phase diagrams in F^- media.* 7
Figure S3. *SEM images of ITQ-56 material*...7
Figure S4. *3D reciprocal lattice and 2D slices h0l, 0kl and hk0 of ITQ-56 reconstructed from the cRED data* 8
Figure S5. *The plots of Rietveld refinement against synchrotron PXRD of the as-made ITQ-56*...9
Figure S6. *Difference Fourier map after the refinement of the ITQ-56 with cRED data.* 9
Figure S7. *^{13}C liquid NMR of OSDA and ^{13}C-MAS-NMR of ITQ-56*..10
Figure S8. *^{19}F-MAS-NMR spectrum.*...10
Figure S9. *Thermogravimetric analysis of as-made ITQ-56.*..11
Figure S10. *Pore sizes in zeolite ITQ-56.* 11
Figure S11. *The structure of ITQ-56 along a and the structure of ITQ26 along c.* 12
Figure S12. *Bloch decay ^{29}Si MAS-NMR spectrum and ^{1}H to ^{29}Si CPMAS NMR of the as-made material of ITQ-56.* 12
Figure S13: *Textural properties of ITQ-56.*...13
Figure S14: *In-situ XRD calcination up to 600ºC.* 13
Table S1. *Lattice energies for interrupted frameworks.*..14
Figure S15. *Structures of ITQ-56 and related hypothetical 22-ring zeolite frameworks deduced from ITQ-26, ITQ-7 and ITQ-21, respectively.* 14

EXPERIMNETAL

Synthesis of OSDA

8.3 g of Memantine (Aladdin), 39.5 g CH_3I (Energy Chemical), 12.81 g K_2CO_3 100ml ethanol are added to flask stirring for 2 days, 19.8 g CH_3I is added, continue to stirring for 2 days more. Then evaporate the ethanol, add 150 ml of chloroform, filtrate off the K_2CO_3. Oil like product can be collect after the evaporation of chloroform. Wash with diethyl ether, large amount of white precipitate is formed, filtrate, and dry overnight. 14.74 g white solid product is obtained (Yield 91%).

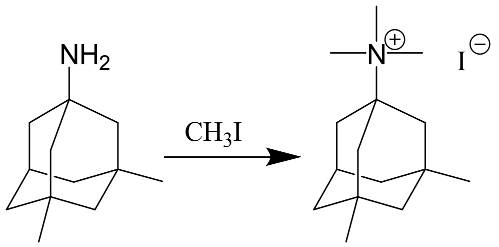

Scheme S1. Synthesis of OSDA molecule

The product is exchanged to the hydroxide form with an anionic exchange Amberlite IRN-78 resin in batch overnight.

Synthesis of ITQ-56 zeolite

The novel zeolite ITQ-56 was obtained after crystallization at 200 °C for one day using the 3,5,N,N,N-pentamethyl-1-adamantammonium hydroxide cation as the OSDA, from a synthesis gel with the following composition: $0.667SiO_2$:$0.333GeO_2$:0.15OSDAOH:$0.15NH_4F$:$3H_2O$. Ludox (SiO_2, 40% Aldrich), SDAOH, GeO_2 (99.998%, Aldrich), $Al(OH)_3$ (58% wt, Al_2O_3), NH_4F (99.99% Aldrich,10% wt water solution), were introduced into 3 ml Teflon vials and stirred while evaporating remaining water until the final desired water content was achieved. Teflon vials were inserted into the multiautoclave and the crystallization was carried out at 200 ºC for one day in static. The samples were characterized by PXRD on a HT X-ray diffractometer.

Structural analysis by transmission electron microscope (TEM) and continuous rotation electron diffraction (cRED).

TEM and cRED investigations were carried out with a JEOL JEM-2100 TEM operated at 200 kV. The powder of ITQ-56 was crushed in an agate mortar, dispersed in ethanol and treated by sonication for 2 minutes. In order to remove the amorphous phase in the sample, the prepared ITQ-56 suspension was decanted and sonicated several times. A droplet of the suspension was then transferred to a copper EM grid covered by a holey carbon film. A cryo-transfer tomography holder (Gatan 914) was used and the EM

sample grid was cooled down to -175° before insertion into the TEM. The temperature was kept at -175° during the entire data collection process. The cRED data were collected using SoPhy Software and a highly sensitive hybrid pixel Timepix Quad camera. During the cRED data collection, the goniometer was tilted continuously with a rotation speed of 0.45° per second and an exposure time for each frame was 0.5 seconds. The cRED datasets were processed using the software XDS developed for X-ray diffraction. The structure of ITQ-56 was solved by direct methods using the software SIR2014, and further refined using software SHELXL-97.

Rietveld refinement.

Synchrotron PXRD data were collected from the *as-made* sample of ITQ-56 at European Synchrotron Radiation Facility (ESRF) in Grenoble (λ= 0.39984 Å). The sample was sealed in a Kapton capillary of 1.00 mm in diameter. Initially, a Pawley fit was performed to optimize unit cell parameters, background and peak shape function. Structure model determined from cRED data was refined against synchrotron PXRD data using TOPAS Academic V5. Data up to 2theta of 18° corresponding to a d-spacing of 1.278 Å were used. The Rietveld refinement was conducted using Pseudo-Voigt (PV) type peak profile function, and the zero shift, capillary shift, unit cell parameters, atomic positions of T and O atoms, silicon and germanium occupancies and atomic displacement parameters were refined. The occupancy of Ge and Si was refined freely during Rietveld refinement for all T sites with the total occupancy (Si+Ge) for each site set to 1.0. The occupancy of the disordered 4-rings was set to 0.5. Geometric restraints were applied for all T-O (1.61 – 1.74 Å) distances and O-T-O (109.5°) angles. Geometric restraints were gradually released during the progress of the refinement. The starting position for the four OSDA molecules were obtained from the difference Fourier map obtained from refinement using cRED data (Figure S5). The position of the molecules were initially refined as a rigid body and later the geometric restraints were gradually released. The fluoride ions were found from the difference Fourier map and their occupancies were refined.

ICP analysis

A 30 mg sample for ICP (Inductively Coupled Plasma) analysis was dissolved in a mixed acid solution 1ml HF (40%, Merck Suprapur®), 1ml HNO_3 (65%, Merck, Emsure®), 3ml HCl (30%, Merck Suprapur®) at room temperature 24h and then diluted to 60g with distillated water and sent to VARIAN715-ES ICP-PLASMA.

CHN analysis

CHN analysis were performed using EuroEA elemental analyser.

Thermogravimetric analysis

Thermogravimetric analysis were performed with a METTLER TOLEDO TGA/SDATA851e, from a room temperature to 800 ºC with a heating rate of 10 ºC/min.

Temperature depended in situ PXRD

Temperature depended *in situ* PXRD was performed in an Anton Parr XRK-900 chamber attached to a PANalytical X'Pert PRO diffractometer to follow the calcination of the sample. The sample was heated from room temperature to 600 ºC with a heating rate of 3 º/min.

Scanning electron microscopy (SEM)

SEM images were acquired using a JEOL JSM-7401F microscope with a below – the – lens (LEI) secondary electron detector and Cold FEG system operated at an accelerating voltage from 1 kV to 2 kV and working distance of 8 mm.

NMR studies

^{1}H and ^{13}C liquid-NMR data were measured for the organocations in solution ($CDCl_3$ as solvent) with a Bruker Avance300 spectrometer. The solid-state MAS-NMR spectra were recorded at a room temperature with a Bruker AV400 spectrometer. ^{1}H to ^{13}C CP (cross-polarized) MAS-NMR spectra were recorded with proton decoupling, with 90º pulse length for ^{1}H of 4.5 ms, a contact time of 3 ms and recycle delay of 3s. ^{29}Si BD (bloch decay) MAS-NMR spectra were measured using pulses of 4 ms corresponding to a flip angle of π/3 radians and recycle delay of 240 s. The ^{1}H to ^{29}Si CP MAS-NMR spectra were recorded using a 90º pulse length for ^{1}H of 4.5 ms, and a contact time of 1 ms and recycle delay of 3s. The ^{13}C and ^{29}Si spectra were recorded using a BL-7 probe with 7 mm diameter zirconia rotors spinning at 5 kHz. ^{19}F MAS-NMR spectra were recorded at 25 kHz spinning speed in a BL2.5 probe using 2.5 mm diameter rotor with a 90º pulse length of 5 ms and a recycle delay of 60s. The ^{13}C, ^{29}Si and ^{19}F spectra were referred to adamantane (CH_2 signal at 38.3 ppm), TMS (0 ppm), and $CFCl_3$ (0 ppm) respectively.

Textural properties

The sample for textural analysis was calcined at 540 ºC (with a heating rate of 2 ºC/min from room temperature) for 6 h in a dry air atmosphere. During the cooling to room temperature, the airflow was substituted by N_2 flow until the sample was sealed in a gas adsorption tube to be degassed. The N_2 adsorption curve was measured on a Micromeritics ASAP 2420, degassing at 400 ºC. The Argon adsorption was measured on a Micromeritics ASAP 2020, after degassing also at 400 ºC.

Energetic studies

Lattice energy

Energy-minimization calculations were performed on pure silica structures by first reducing the symmetry to *P*1 using the General Utility Lattice Program (GULP) package1. The long-range coulombic interactions were evaluated by the Ewald method2, whereas the short-range interactions were obtained using the direct summation. The breathing core-shell model was used throughout to describe the polarizability of asymmetric atoms using the SLC potential,23 and the rational function optimization method was used, 24 as implemented in GULP. The structures were verified to contain only positive

phonon frequencies. Oxygen and hydrogen atoms belonging to silanol groups were modeled as a core only with the charges modified to be -1.426 and 0.426, respectively. The same Buckingham potential was used for all oxygen atoms but the Morse potential was used to model the O-H bond. The lattice energies of the optimized structures were evaluated relative to α-quartz. A hypothetical, fully connected ITQ-56 with additional *d4r*s was constructed. Frameworks constituting the same building components as ITQ-56, ITQ-7 (ISV), ITQ-21, and ITQ-26 (IWS) were modified to create silanol containing interrupted frameworks. Supercells were carefully constructed as to mimic ITQ-56 framework in which the interruption occurs every other column as shown in Fig. S13. Every *d4r* types were removed for creating the hypothetical structures.

Stabilization energy

ITQ-56 and its hypothetic fully-connected zeolite frameworks were both geometry optimized using the SLC potential as previously described. The optimized models were used for single-point calculations using the Dreiding force field[1] to estimate their energies (E_z).[1] The memantine molecule was put in a 30 x 30 x 30 Å^3 unit cell to relax and evaluate their final energy (E_{SDA}) using the charge-less Dreiding force field to avoid any dispersion forces between the molecules. The memantine molecules were then introduced into the optimized ITQ-56 framework according the Rietveld refined sites against the synchrotron PXRD data. For the hypothetic framework, two molecules per 22-ring channel were removed considering that only two memantine molecules exist along the other 12-ring channels. Molecular dynamics (MD) simulation was then performed using the NVT ensemble on the zeolite-OSDA complex while fixing the framework atoms to find optimal positions of the OSDAs at 343 K using the Dreiding force field for 1 ps. The final coordinates from the MD simulation were further optimized to obtain the energy of the zeolite-OSDA complex (E_{Z+SDA}). The stabilization energy was calculated by $E_{Z+SDA} - nE_{SDA} - E_z$, where n is the number of memantine molecules per unit cell.

FIGURES

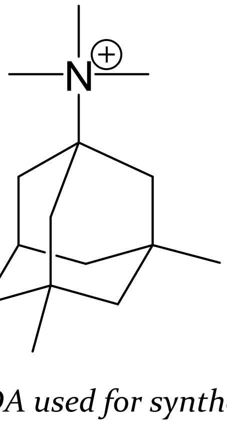

Figure S1: *Structure of OSDA used for synthesizing the ITQ-56 zeolite.*

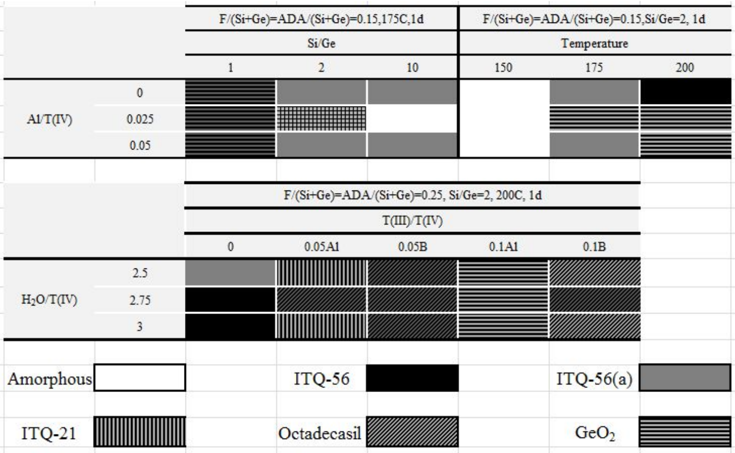

(a): poor crystallinity

Figure S2: *Phase diagrams in F^- media.*

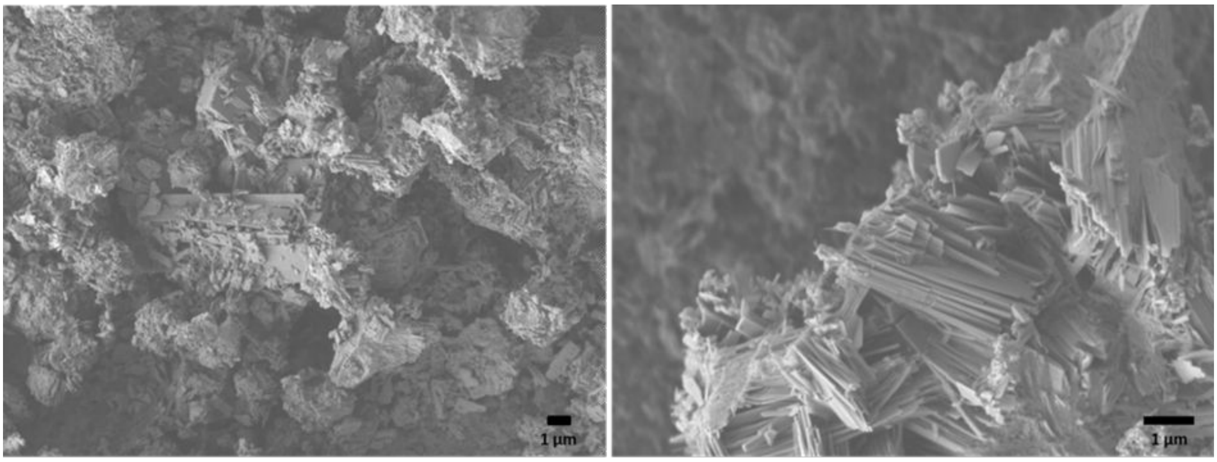

***Figure S3**. SEM images of ITQ-56 material revealing plate-like crystals surrounded by a large amount of the amorphous material.*

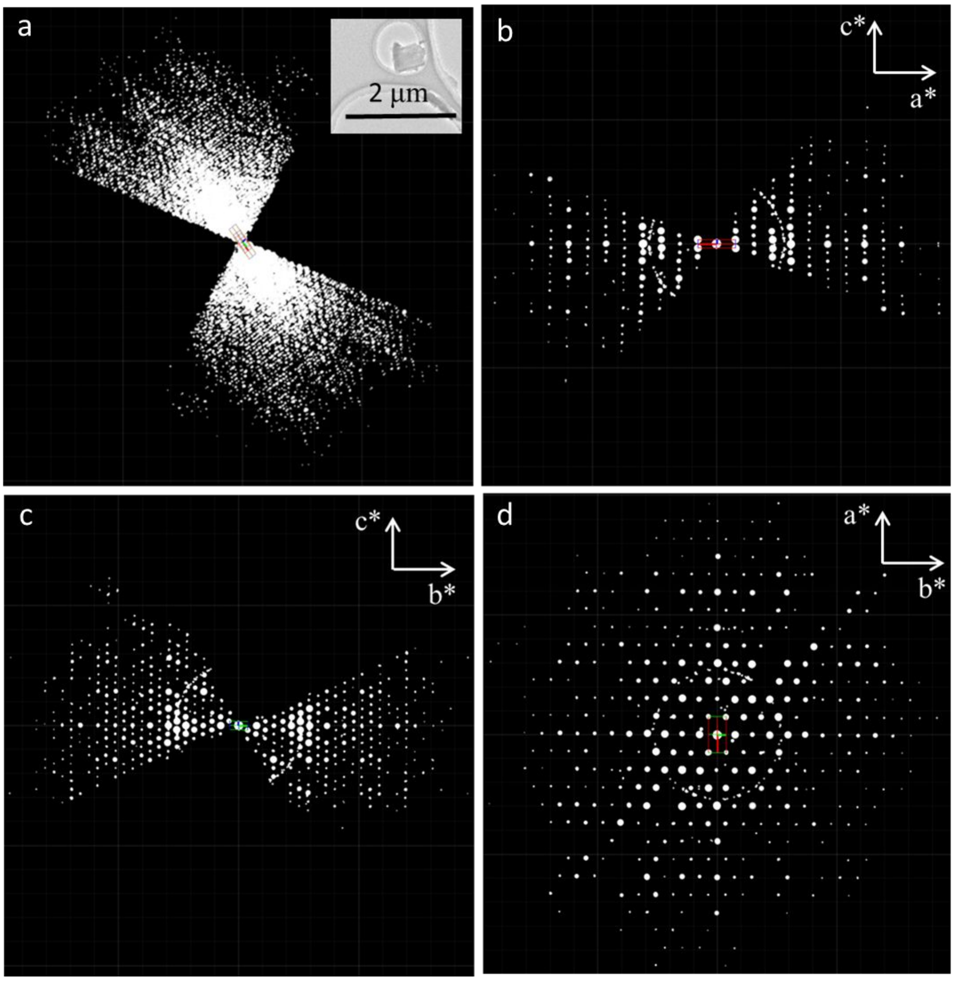

Figure S4. *a) 3D reciprocal lattice of ITQ-56 reconstructed from the cRED data that were obtained from crystal shown in the corner. 2D slices h0l, 0kl and hk0 are shown in b), c) and d) respectively. Reflection conditions can be observed and are as follow: hkl: h + k + l =2n, hk0: h + k =2n, h0l: h + l =2n, 0kl: k + l =2n, h00: h = 2n, 0k0: k =2n.*

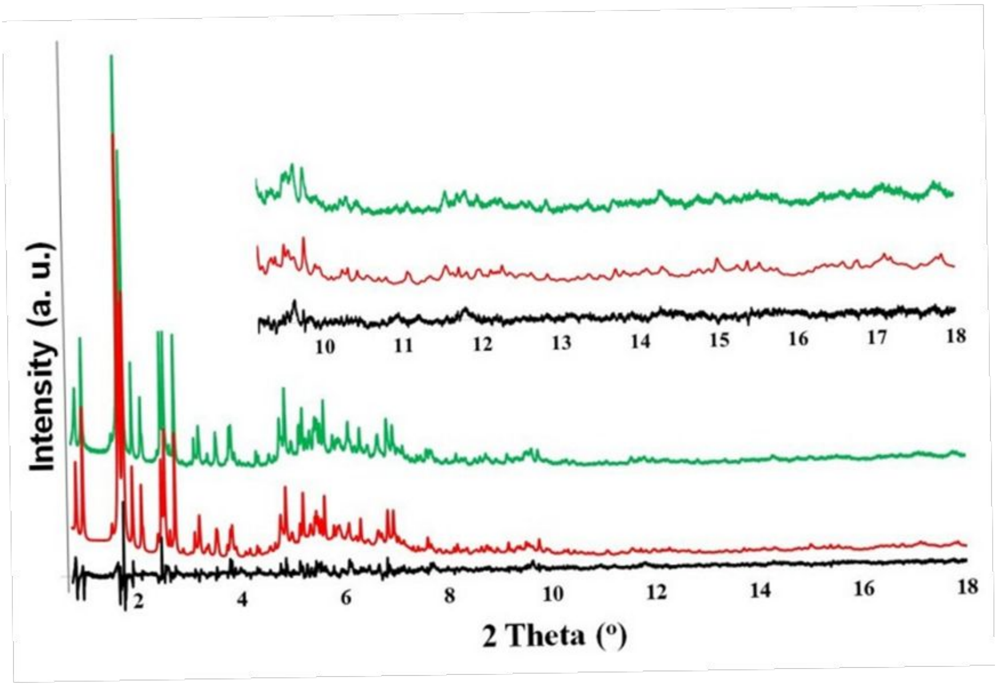

Figure S5. *The plots of Rietveld refinement against synchrotron PXRD of the as-made ITQ-56, the part from 9 to 18° 2theta is enlarged by a factor of 3. The curves from top to bottom are observed (green), calculated (red) and difference (black) profiles respectively (λ= 0.39984 Å).*

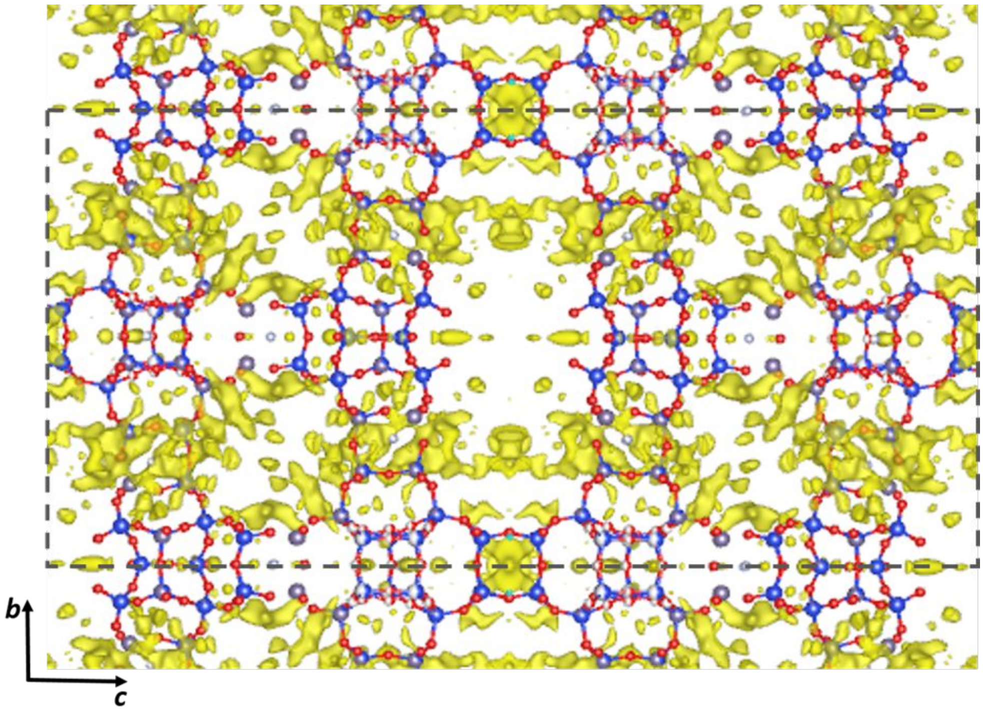

Figure S6. *Difference Fourier map after the refinement of the ITQ-56 against cRED data.*

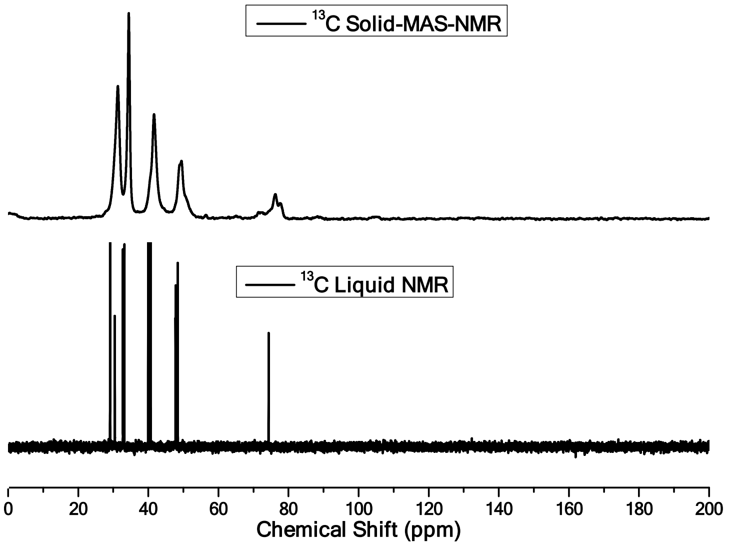

Figure S7. *13C liquid NMR (bottom) of OSDA and 13C-MAS-NMR(top) of ITQ-56 spectra comparison*

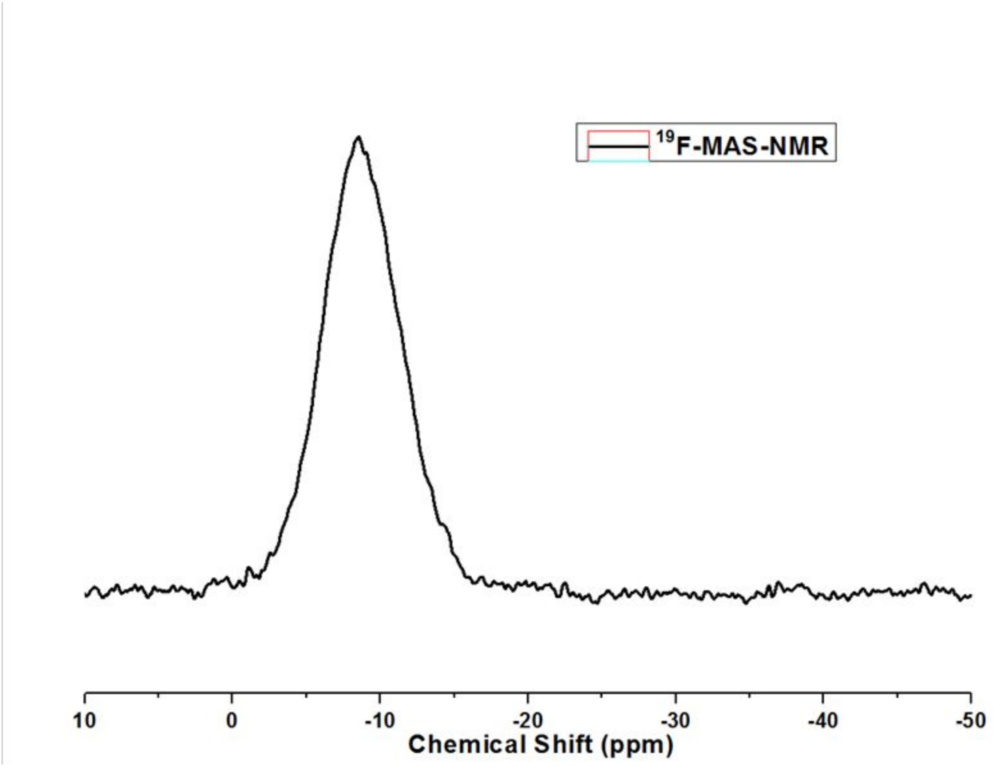

Figure S8. *19F-MAS-NMR spectrum.*

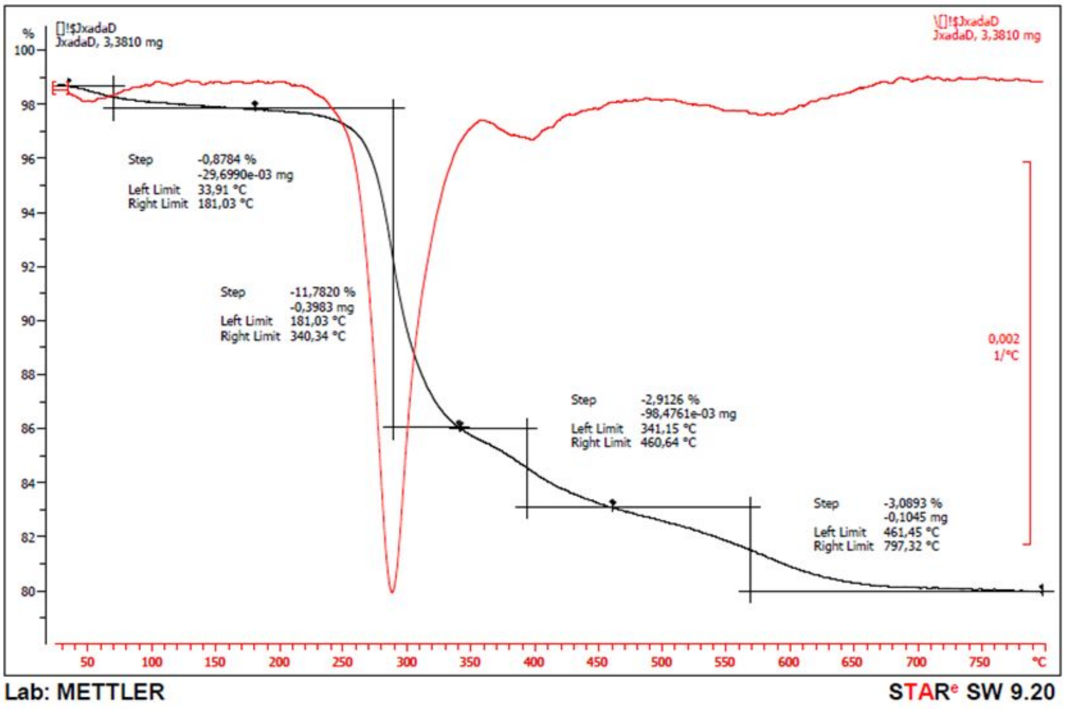

Figure S9. *Thermogravimetric analysis of as-made ITQ-56.*

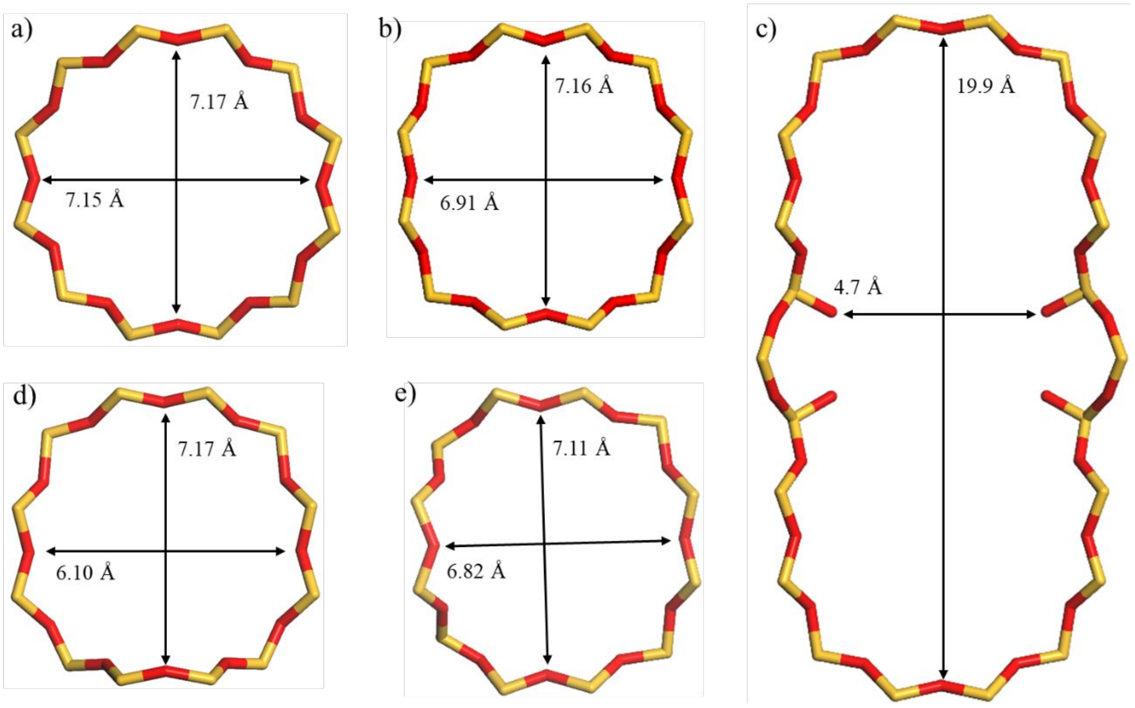

Figure S10. *Pore sizes in zeolite ITQ-56. a) 12-ring along [100], b) 12-ring along [010], c) 22-ring along [100], d) 12-ring along <110> and e) 12-ring along <101>. The marked distances are after subtracting two van der Waals radii for oxygen - 2.7 Å.*

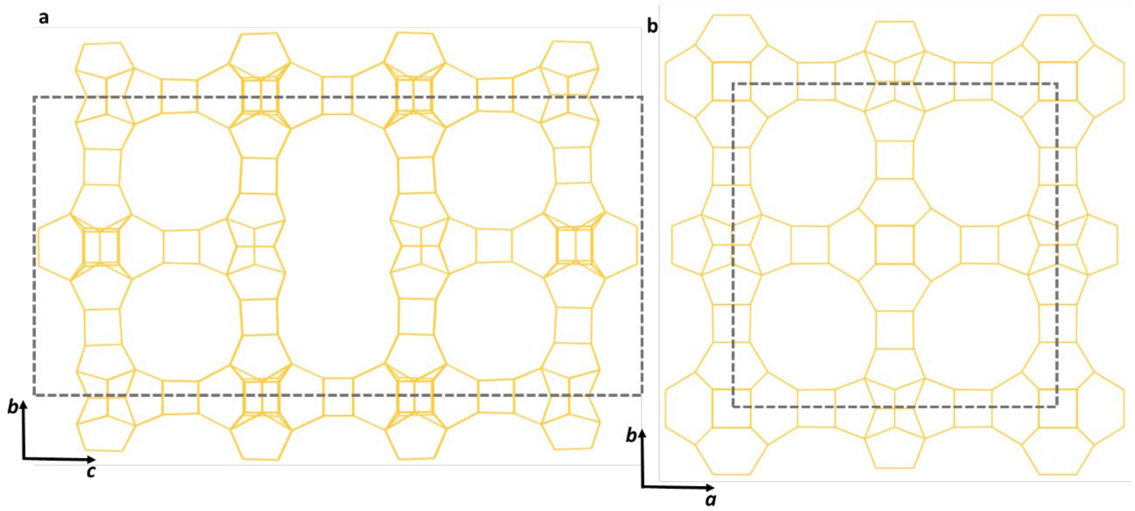

Figure S11. *a) The structure of ITQ-56 along a and b) the structure of ITQ-26 along c.*

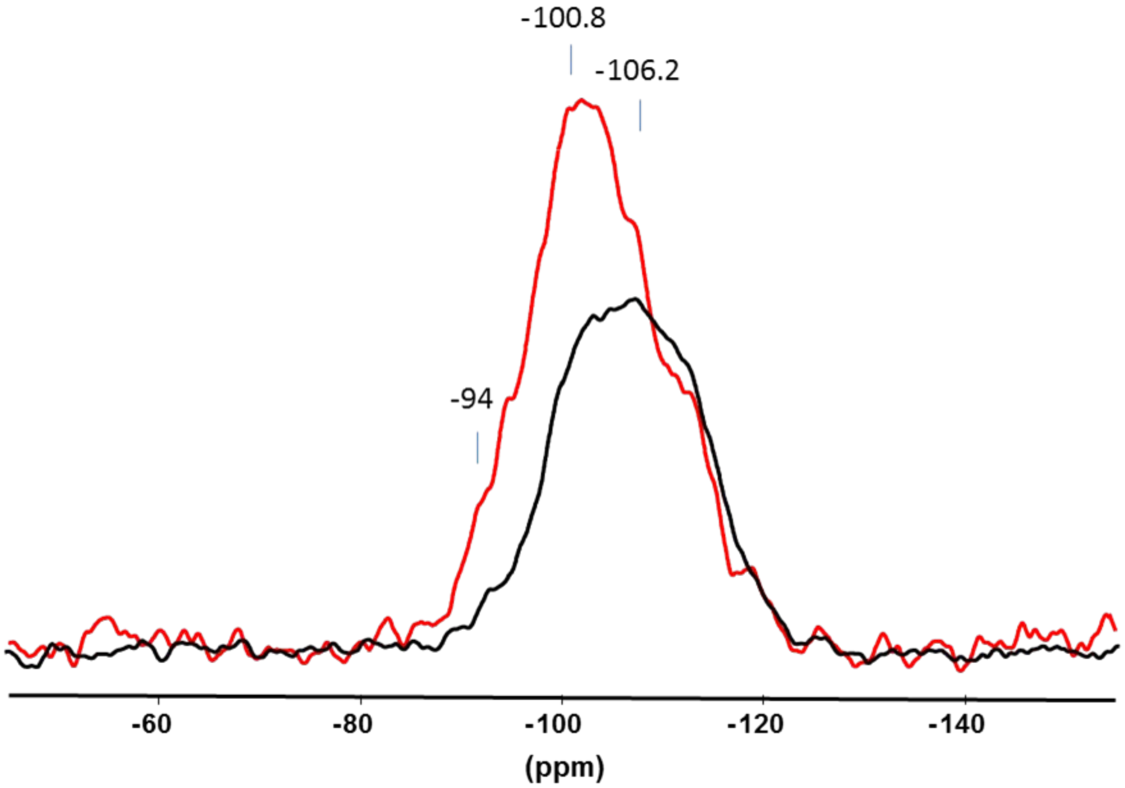

Figure S12. *Bloch decay ^{29}Si MAS-NMR spectrum (black) and ^{1}H to ^{29}Si CPMAS NMR (red) of the as-made material of ITQ-56.*

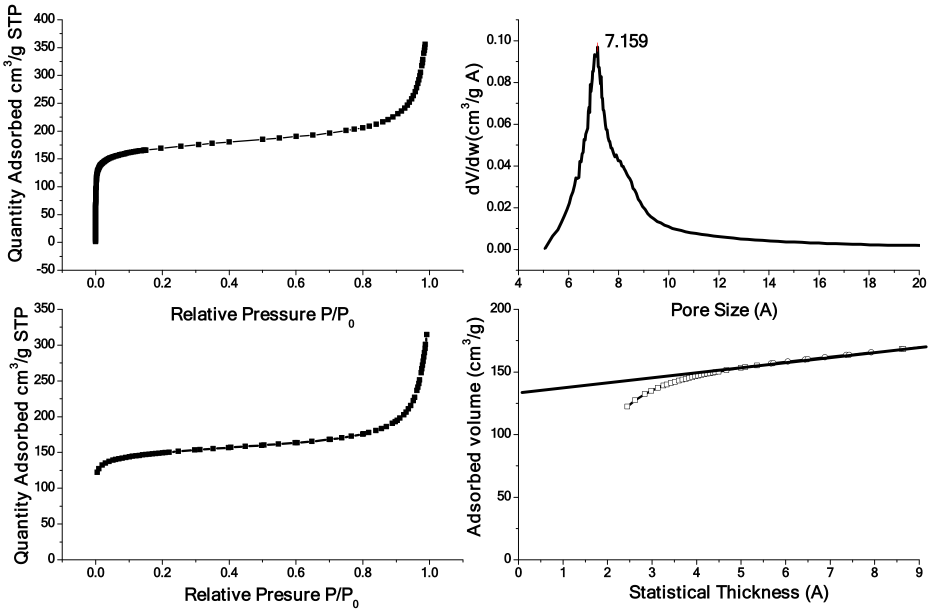

Figure S13: *Textural properties of ITQ-56. Top: High resolution Ar adsorption isotherm at 87K (left) and pore diameter distribution determined from the Ar isotherm (right); bottom: Adsorption N_2 isotherms at 77K (left) and t-plot calculated from the N_2 adsorption isotherm (right).*

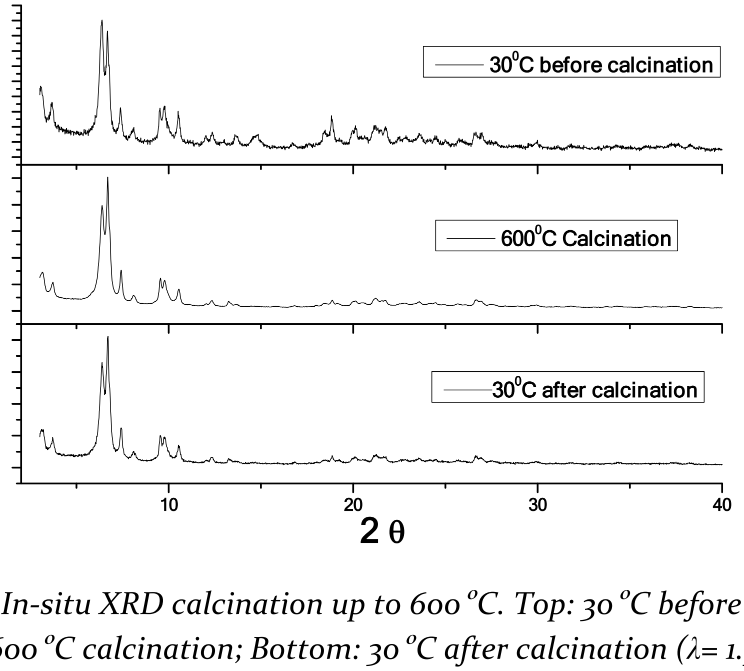

Figure S14: *In-situ XRD calcination up to 600 °C. Top: 30 °C before calcination; Middle:600 °C calcination; Bottom: 30 °C after calcination (λ= 1.5406 Å).*

Table S1. *Lattice energies for interrupted frameworks.*

Zeolite	*Energy relative to Quartz (kJ/molSi)*
ITQ-56	*10.41*
ITQ-26_OH	*12.95*
ITQ-7_OH	*13.10*
ITQ-21_OH	*11.91*

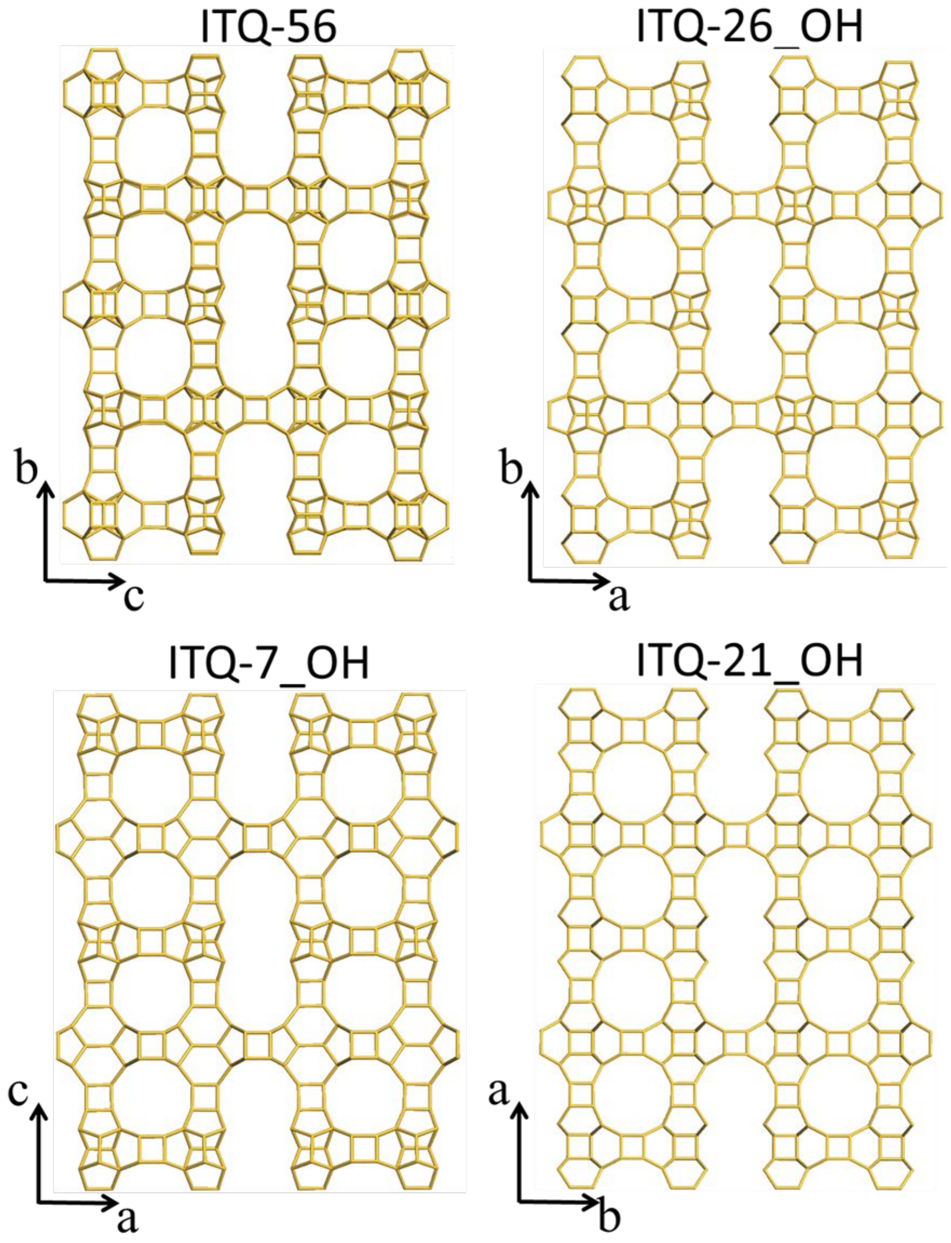

Figure S15*. Structures of ITQ-56 and related hypothetical 22-ring zeolite frameworks deduced from ITQ-26, ITQ-7 and ITQ-21, respectively. Ordered vacancies of d4r units are introduced in a similar way as found in ITQ-56.*
